# Robotic Ureteroureterostomy for Treatment of a Proximal Ureteric Stricture

**DOI:** 10.1590/S1677-5538.IBJU.2015.0249

**Published:** 2016

**Authors:** Hiury S. Andrade, Jihad H Kaouk, Homayoun Zargar, Peter A. Caputo, Oktay Akca, Daniel Ramirez, Riccardo Autorino, Mark Noble, Robert J. Stein

**Affiliations:** Center for Robotic and Image Guided Surgery, Glickman Urological and Kidney Institute, Cleveland Clinic, Cleveland, OH, USA

## INTRODUCTION

Ureteral stricture in the proximal ureter is a rare condition with few effective treatment options. The purpose of this video is to demonstrate a simplified robotic ureteroureterostomy technique.

## CASE

A 27-year old female was diagnosed with a left 5mm ureteral stricture, 3 cm from the UPJ. Patient had the history of ureteroscopy for a 1.6 × 1.0 cm stone 13 months prior. After unsuccessful attempts of endoscopic treatments including laser endoureterotomy and indwelling ureteral stent placement, she underwent robotic ureteroureterostomy.

The patient was placed in 60° flank position with the ipsilateral arm positioned on the side of the patient. Five ports were placed in a straightline configuration lateral to the rectus muscle and the robot was docked perpendicular to the patient torso. The ureter was dissected and the stricture area identified intraoperatively using the surrounding scar tissue and proximal dilatation as clues. The ureter was transected and spatulated. In order to minimize tension, two peri-ureteral sutures were performed prior the ureteroureterostomy anastomosis.

## RESULTS

The operative time was 300 minutes and the estimated blood loss was 50 mL. There were no intra or postoperative complications. The double J stent was removed after 4 weeks and no recurrence was noted after 27 months. The 1-year postoperative renal scintigraphy showed a split renal function of 43.3% in the treated side, with a T1/2 of 7.0 minutes.

## CONCLUSION

Robotic ureteroureterostomy is a suitable and feasible alternative for the treatment of short proximal ureteral strictures where endoscopic modalities have been unsuccessful.

## ARTICLE INFO

**Available at:** **http://www.intbrazjurol.com.br/video-section/Andrade_1041_1042/**

**Int Braz J Urol. 2016; 42 (Video #9): 1041-2**

